# Long-term impact of myocardial inflammation on quantitative myocardial perfusion—a descriptive PET/MR myocarditis study

**DOI:** 10.1007/s00259-023-06314-0

**Published:** 2023-07-01

**Authors:** Ronny R. Buechel, Domenico Ciancone, Adam Bakula, Elia von Felten, Gian-Andrea Schmidt, Dimitri Patriki, Christoph Gräni, Andreas Wahl, Robert Manka, Bettina Heidecker, Dominik C. Benz, Andreas A. Giannopoulos, Aju P. Pazhenkottil, Philipp A. Kaufmann

**Affiliations:** 1 Department of Nuclear Medicine, Cardiac Imaging, University and University Hospital Zurich, Ramistrasse 100, NUK A 12, 8091 Zurich, Switzerland; 2 Department of Cardiology, Inselspital, Bern University Hospital, University of Bern, Bern, Switzerland; 3 Department of Cardiology, University and University Hospital of Zurich, Zurich, Switzerland; 4 Diagnostic and Interventional Radiology, University and University Hospital Zurich, Zurich, Switzerland; 5 Department of Cardiology, Campus Benjamin Franklin, Charité - Universitätsmedizin Berlin, corporate member of Freie Universität Berlin und Humboldt-Universität zu Berlin, Berlin, Germany

**Keywords:** Myocarditis, Inflammation, Cardiac magnetic resonance, Positron emission tomography, Myocardial blood flow, Perfusion

## Abstract

**Purpose:**

Whether myocardial inflammation causes long-term sequelae potentially affecting myocardial blood flow (MBF) is unknown. We aimed to assess the effect of myocardial inflammation on quantitative MBF parameters, as assessed by 13N-ammonia positron emission tomography myocardial perfusion imaging (PET-MPI) late after myocarditis.

**Methods:**

Fifty patients with a history of myocarditis underwent cardiac magnetic resonance (CMR) imaging at diagnosis and PET/MR imaging at follow-up at least 6 months later. Segmental MBF, myocardial flow reserve (MFR), and 13N-ammonia washout were obtained from PET, and segments with reduced 13N-ammonia retention, resembling scar, were recorded. Based on CMR, segments were classified as remote (*n* = 469), healed (inflammation at baseline but no late gadolinium enhancement [LGE] at follow-up, *n* = 118), and scarred (LGE at follow-up, *n* = 72). Additionally, apparently healed segments but with scar at PET were classified as PET discordant (*n* = 18).

**Results:**

Compared to remote segments, healed segments showed higher stress MBF (2.71 mL*min^−1^*g^−1^ [IQR 2.18–3.08] vs. 2.20 mL*min^−1^*g^−1^ [1.75–2.68], *p* < 0.0001), MFR (3.78 [2.83–4.79] vs. 3.36 [2.60–4.03], *p* < 0.0001), and washout (rest 0.24/min [0.18–0.31] and stress 0.53/min [0.40–0.67] vs. 0.22/min [0.16–0.27] and 0.46/min [0.32–0.63], *p* = 0.010 and *p* = 0.021, respectively). While PET discordant segments did not differ from healed segments regarding MBF and MFR, washout was higher by ~ 30% (*p* < 0.014). Finally, 10 (20%) patients were diagnosed by PET-MPI as presenting with a myocardial scar but without a corresponding LGE.

**Conclusion:**

In patients with a history of myocarditis, quantitative measurements of myocardial perfusion as obtained from PET-MPI remain altered in areas initially affected by inflammation.

**Graphical abstract:**

CMR = cardiac magnetic resonance; PET = positron emission tomography; LGE = late gadolinium enhancement

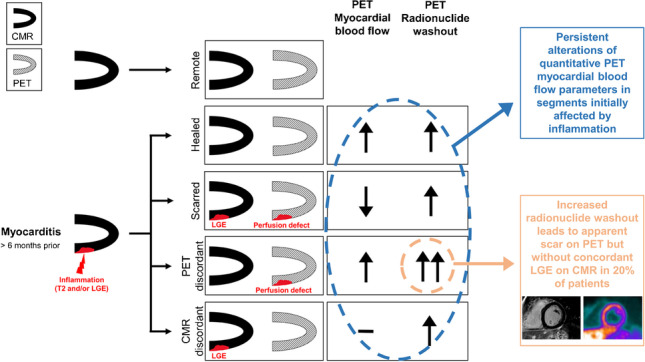

**Supplementary Information:**

The online version contains supplementary material available at 10.1007/s00259-023-06314-0.

## Introduction

Myocarditis is an entity that clinically manifests in very diverse ways, rendering the true incidence of the disease difficult to assess. However, it is not uncommon and has been found in up to 9% of routine autopsy cases and in up to 30% of those performed for unexplained sudden cardiac death in young individuals [1, 2]. It is an inflammatory disease of the myocytes often caused by a viral infection that produces myocardial necrosis and triggers an immune response to eliminate the infectious agent. While the attracted inflammatory cells are crucial to the inflammatory clearance of pathogens, the production of pathological mediators (e.g., TGF-b) during this process may lead to further injury of cardiomyocytes and induce fibrosis [3]. A vast amount of literature exists on the etiology, pathogenic mechanisms, natural course, prognosis, and means to diagnose acute viral myocarditis [4, 5], but very little is known about the long-term effects of inflammation on the affected myocardium, and, in particular, on the microcirculatory system. A number of smaller studies and case series have shown that acute myocarditis may lead to perfusion defects identifiable on 201-thallium or 99 m-technetium sestamibi/tetrofosmin single-photon-emission-computed-tomography myocardial perfusion imaging (SPECT-MPI) [6–8], and some experimental studies suggested disturbances in the coronary microcirculation and a reduction in myocardial flow reserve (MFR) [9, 10]. For example, Klein et al., using an argon gas chromatographic method, have suggested that MFR in patients with biopsy-proven myocarditis was significantly lower than that in a control group [11]. However, no data are available neither on the reversibility of this phenomenon nor on the long-term effects with regard to myocardial blood flow (MBF).

The present study aimed to evaluate whether quantitative MBF parameters, as assessed by the gold-standard positron emission tomography myocardial perfusion imaging (PET-MPI), remain permanently altered in myocardial regions affected by inflammation in patients with a history of myocarditis.

## Methods

### Study design

Fifty patients with a history of clinically suspected and cardiac magnetic resonance imaging (CMR)-confirmed myocarditis deemed to be caused by a viral infection [12] and diagnosed at least 6 months prior were prospectively included between 04/18 and 12/19. Exclusion criteria were defined as any contraindications against CMR (e.g., implanted cardiac devices, claustrophobia, allergy against gadolinium-based contrast agents [GBCA], severe renal impairment), against adenosine (e.g., asthma or higher-grade atrioventricular block), or contraindications for PET (e.g., pregnancy or breastfeeding). Furthermore, patients with concomitant coronary artery disease (CAD) and known hypertrophic or infiltrative cardiomyopathies were excluded. The study protocol was approved by the local ethics committee (BASEC-Nr. 2018-00170), and all patients provided written informed consent.

### Baseline CMR imaging and analysis

CMR datasets were acquired on a 1.5- or 3.0-Tesla scanner (Philips Achieva, Intera or Ingenia, Best, the Netherlands or Siemens Aera or Skyra, Erlangen, Germany) using an electrocardiogram-gated breath-hold protocol. The diagnosis of myocarditis was based on cine-CMR, T2-weighted imaging, and T1-weighted late gadolinium enhancement (LGE) imaging. LGE images were generated 10 min after the intravenous administration of a GBCA. For the present study, all datasets were analyzed with commercially available software (cvi42, release 5.11.3, Circle Cardiovascular Imaging, Calgary, AB, Canada). Myocardial LGE and/or T2 hyperintensities of the left ventricle (LV) were visually identified, and the software’s tissue characterization module was used to assess enhancement in myocardial segments with LGE and/or T2 hyperintensities using a threshold of 5 and 2 standard deviations (SD) over remote myocardium, respectively. Polar plots based on the American Heart Association (AHA) 16-segment LV model were then generated, allowing for a direct segmental comparison between baseline and follow-up CMR and PET-MPI. For calculation of LV and right ventricular (RV) end-diastolic volumes (LVEDV and RVEDV), LV and RV end-systolic volumes (LVESV and RVESV), LV mass index (indexed by body surface area, LVMi), and LV and RV ejection fractions (LVEF and RVEF), endocardial contours were traced in short-axis views.

### Follow-up hybrid PET/CMR imaging and analysis

**Fig. 1:**
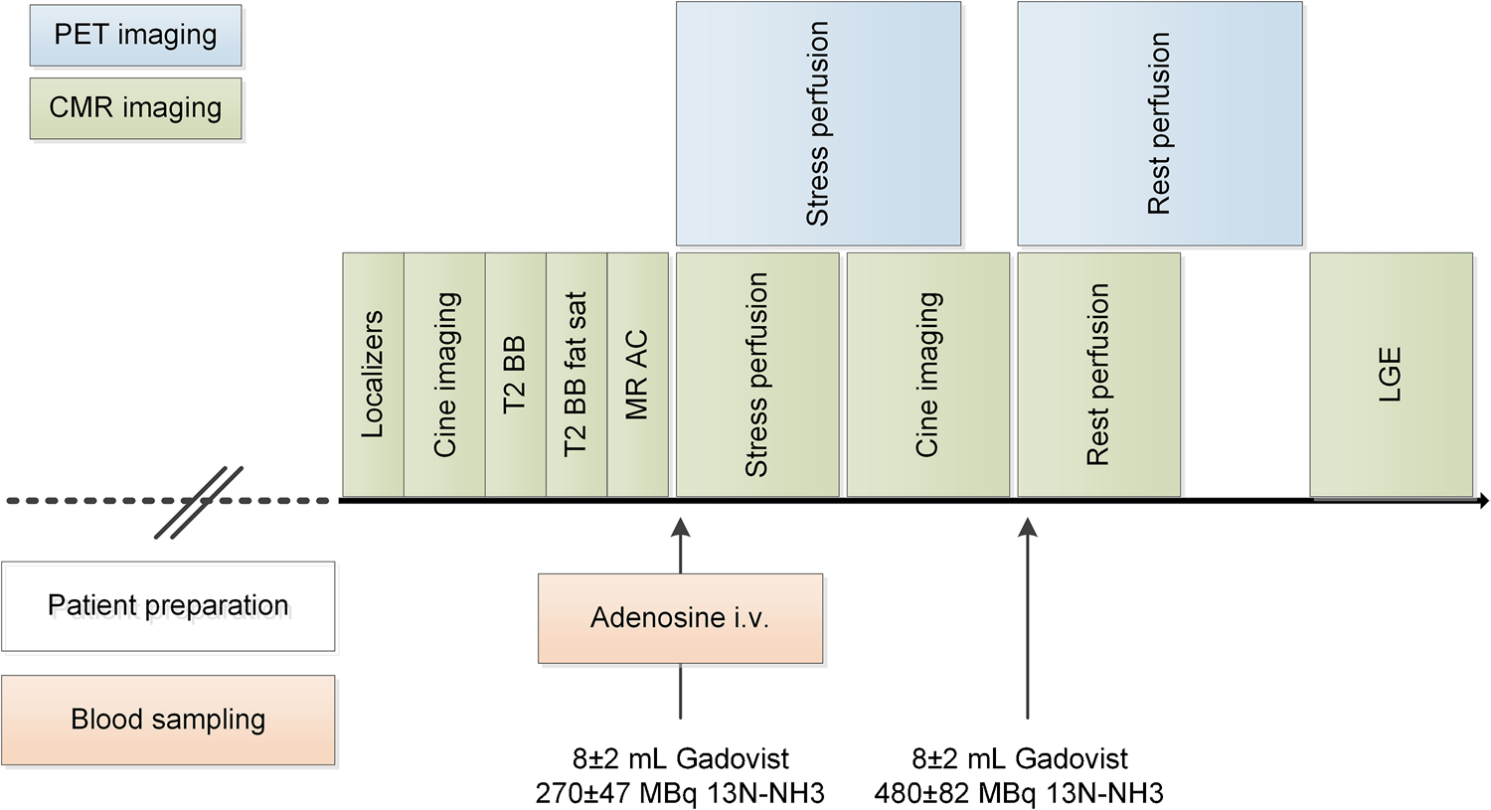
Hybrid PET/MR imaging acquisition protocol employed at follow-up

PET and CMR datasets were acquired on a hybrid PET/MR device incorporating a 3.0-Tesla MR and a latest-generation PET scanner (Signa PET/MR, GE Healthcare, Waukesha, WI, USA). All patients refrained from caffeine intake for at least 12 h before the examination. The stress protocol consisted of 6 min of adenosine infusion (140 μg*kg-1*min-1). A body mass index-adapted dose of 13N-ammonia (i.e., 200–600 megabecquerels (MBq)) and a weight-adapted dose of GBCA (Gadovist, Bayer AG, Zurich, Switzerland; 0.1 mmol/kg) were simultaneously injected after 3 min into adenosine stress. Dynamic PET data acquisition consisted of 21 frames (i.e., 9 × 10-s, 6 × 15-s, 3 × 20-s, 2 × 30-s, and 1 × 120-s), followed by an electrocardiogram (ECG)-gated acquisition over 10 min. Resting perfusion imaging was performed thereafter using an identical protocol. All PET data were acquired in 3D mode and reconstructed using time-of-flight reconstruction with VUE Point FX (2 iterations and 16 subsets) and a 5 mm Hanning filter. Standard Dixon-based maps were used for attenuation correction. CMR datasets were acquired in parallel using an ECG-gated breath-hold protocol, and LGE images were generated 10 min after the administration of GBCA. The PET/MR imaging acquisition protocol is depicted in Fig. 1.

Quantitative MBF (in mL × min^−1^ × g^−1^) was obtained from stress and rest scans and calculated and analyzed using QPET (Version 2017.7, Cedars-Sinai Medical Center, Los Angeles, CA, USA) using a simplified 2-compartment model. Rest MBF (rMBF), stress MBF (sMBF), and myocardial flow reserve (MFR, calculated as sMBF over rMBF) were recorded per patient and per segment. rMBF was adjusted by the rate-pressure product. Additionally, the K2 constant (i.e., 13N-ammonia washout rate, /min) was calculated for every patient and segment for rest (rK2) and stress (sK2) using PMOD version 3.7 (PMOD Technologies, Zurich, Switzerland). Polar plots based on the AHA 16-segment LV model were generated to display the results of the quantitative analysis. Patients and segments with myocardial scar were defined as having a visually perceivable relative reduction in 13N-ammonia retention as per clinical routine. CMR analysis was performed in an identical manner as the baseline datasets.

### Laboratory analysis

The following routine laboratory parameters were recorded at baseline and at follow-up: high-sensitivity troponin T (Troponin T-hs), creatine kinase (CK), myoglobin, NT-proBNP (N-terminal pro-B-type natriuretic peptide), C-reactive protein (CRP), and leukocyte count.

### Segmental analysis

Based on the hypothesis of this study that myocardial perfusion remains permanently altered after inflammation, we placed a special focus on myocardial segments that were affected by inflammation at baseline. A combination of segmental T2 hyperintensity and/or with LGE at baseline but without LGE at follow-up was considered a sign of inflammation. This methodology is based on the evidence that LGE in the acute phase of myocarditis is not necessarily synonymous with irreversible damage, particularly if it resolves over time [13, 14]. We defined five groups of segments for further analysis: (group 1) remote segments (i.e., without any signs of inflammation at baseline and no LGE at follow-up), (group 2) healed segments (i.e., with signs of inflammation at baseline but no LGE at follow-up), (group 3) scarred segments (i.e., with signs of inflammation at baseline and with persistent LGE at follow-up), (group 4) PET discordant segments (i.e., with signs of inflammation at baseline and with reduced 13N-ammonia retention resembling scar at follow-up PET but no corresponding LGE at CMR), and (group 5) CMR discordant segments (i.e., with signs of inflammation at baseline and with persistent LGE at follow-up but no corresponding reduction in 13N-ammonia retention).

### Statistical analysis

Numbers and percentages were calculated for categorical data and means and SD or median and interquartile range (IQR) for continuous data, as appropriate. Statistical analysis was performed using SPSS Statistics (Version 26, IBM, Armonk, NY, USA). The Shapiro-Wilk test was applied to assess the normality of distribution. Correlations between continuous variables were assessed using Spearman’s analysis. A comparison between two continuous variables with normal and non-normal distributions between different groups of patients or segments was performed using the Student’s *t*-test or Mann-Whitney *U* test, respectively, and with the Kruskal-Wallis test with post hoc pairwise comparison for comparison of multiple groups. We applied the Benjamini-Hochberg procedure to correct for multiple comparisons and to control the false discovery rate (FDR) at 5% [15]. Accordingly, only *p*-values < 0.05 and at a level below the critical *k*-value were considered to indicate statistical significance. Otherwise, two-sided *p*-values < 0.05 were considered significant.

## Results

Patients’ demographics and clinical characteristics are provided in Table 1. The median interval between diagnosis of myocarditis and follow-up was 25 months (IQR 14–41 months, range 6–86 months). Of note, at follow-up, no patients presented with evidence of ongoing myocardial inflammation (i.e., no typical symptoms, myocardial edema, pericardial effusion, or elevated blood markers suggestive of inflammation). CAD was definitely excluded at the time of diagnosis by coronary angiography or myocardial perfusion imaging in 39 (78%) patients and deemed very unlikely due to a typical history and young age (28.3 ± 7.5 years) in the remainder. The median time interval between medical presentation and baseline CMR was 4 days (IQR 2–23).

**Table 1:** Demographics and clinical characteristics at follow-up and laboratory parameters at baseline and follow-up (*n* = 50)

Age, years	38.7 ± 15.2		
Male gender	43 (86%)		
BMI, kg/m^2^	25.6 ± 3.1		
Cardiovascular risk factors			
Arterial hypertension	9 (18%)		
Dyslipidemia	5 (10%)		
Active smoking	14 (28%)		
Past smoking	10 (20%)		
Diabetes	0 (0%)		
Family history of CAD	8 (16%)		
Cardiovascular medication			
None	39 (78%)		
Platelet inhibitors	1 (2%)		
Beta-blockers	3 (6%)		
ACEI/ARB	9 (18%)		
Statin	1 (2%)		
Cardiac symptoms			
None	43 (86%)		
Typical angina	0 (0%)		
Atypical angina	3 (6%)		
Non-anginal chest pain	4 (8%)		
Dyspnea	0 (0%)		
Laboratory parameters			
	Follow-up	Baseline^1^	Reference range
Troponin T-hs, ng/L	5.0 [5.0–7.0]	413.0 [203.0–1100.80]	< 14
CK, U/L	129.0 [100.8–192.5]	269.0 [175.0–508.80]	< 190
Myoglobin, µg/L	26.5 [21.0–36.8]	46.0 [29.3–66.0]	28–72
NT-proBNP, ng/L	25 [18–44]	228.0 [68.5–703.0]	< 85.8
CRP, mg/L	0.8 [0.6–1.3]	25.8 [8.3–56.3]	< 5
Leukocyte count, G/L	5.6 [5.2–6.1]	7.3 [5.7–10.3]	3.0–9.6

Values given are absolute numbers and percentages in parentheses, mean ± SD or median and IQR in square brackets

^1^Laboratory available at baseline in 45 (90%) of patients

### Imaging findings

An overview of CMR and 13N-ammonia PET-MPI findings is given in Table 2. Functional and volumetric CMR parameters did not differ significantly between baseline and follow-up, with LVMi showing a trend towards lower values at follow-up.

**Table 2:** Imaging findings per patient at baseline and/or at follow-up (*n* = 50)

	Baseline	Follow-up	*p*-value	*k*-value
CMR				
LVEDV, mL	159 ± 35	159 ± 32	0.708	0.0050
LVESV, mL	69 ± 21	69 ± 16	0.299	0.0036
LVEF, %	59 ± 7	57 ± 7	0.103	0.0021
LVMi, g/m^2^	65 ± 17	61 ± 14	0.035	0.0007
RVEDV, mL	167 ± 37	157 ± 39	0.078	0.0029
RVESV, mL	75 ± 24	70 ± 22	0.115	0.0014
RVEF, %	56 ± 7	57 ± 6	0.705	0.0043
13N-ammonia PET				
rMBF, mL × min^−1^ × g^−1^		0.66 [0.55–0.83]		
sMBF, mL × min^−1^ × g^−1^		2.27 [1.84–2.79]		
MFR		3.43 [2.64–4.19]		
rK2, /min		0.23 [0.17–0.28]		
sK2, /min		0.49 [0.35–0.64]		
LVEF at rest, %		59 ± 8		
LVEF post-stress, %		58 ± 7		

Values given are mean ± SD or median and IQR in square brackets

**Fig. 2:**
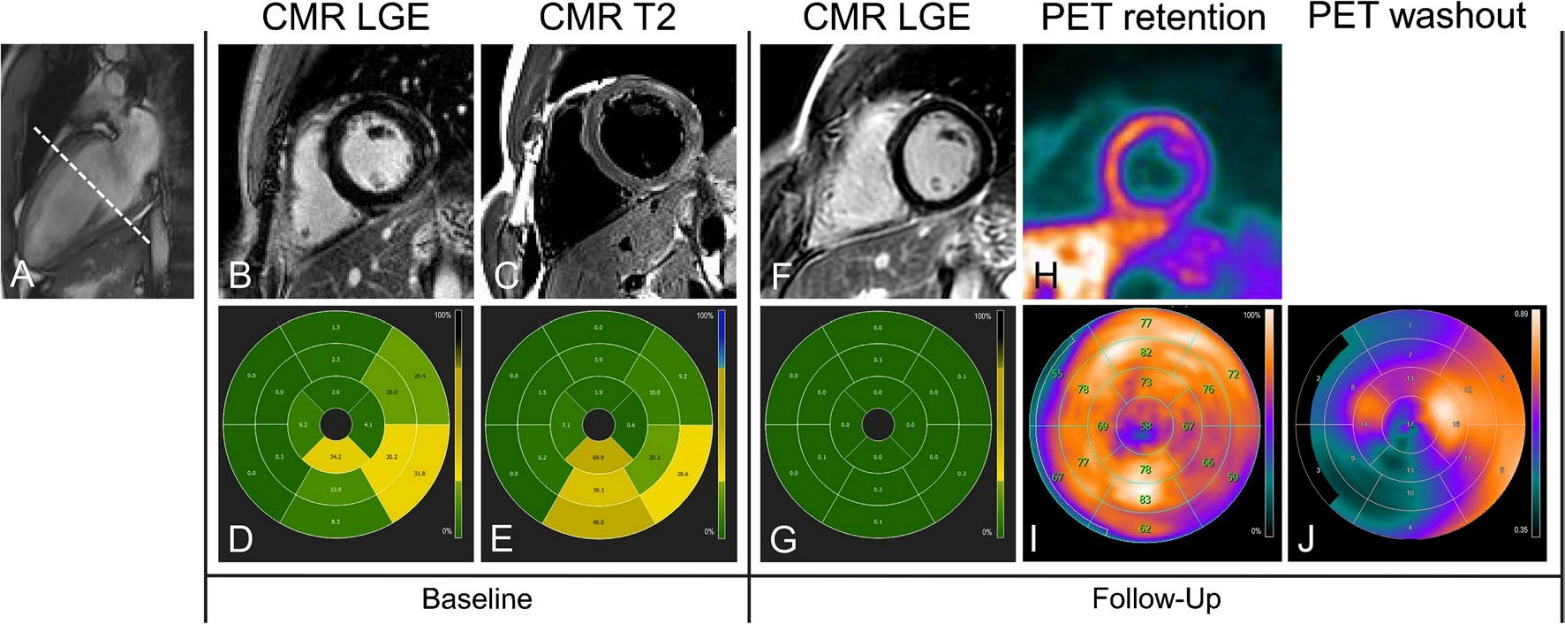
An imaging study of a 22-year-old male with a history of myocarditis. The dotted line in (**A**) depicts the cross-section of the selected slices provided in the top row. CMR at baseline revealed patchy and subepicardial LGE in the anterolateral, lateral, inferolateral, and inferior wall of the basal to mid-ventricular LV myocardium (**B**) with areas of T2 hyperintensity in the same locations (**C**). Polar plots depicting segmental percent enhancement of LGE (**D**) and T2 (**E**) provide an overview of the affected segments. PET/MR was performed 32 months later, with CMR revealing no residual LGE in any segments (**F**, **G**). By contrast, 13N-ammonia PET showed reduced radionuclide retention at rest in the inferolateral basal and in the lateral LV myocardium (**H**, **I**). In parallel, the 13N-ammonia washout rate (**J**) was increased in the lateral and inferolaterobasal LV walls

At baseline CMR, 49 (98%) patients presented with at least one segment with LGE, and 29 (58%) patients presented with at least one segment with T2 hyperintensity. None of the patients showed persistent T2 hyperintensity at follow-up. Persistent LGE, resembling scarred myocardium at follow-up, was found in CMR in 45 (90%) patients. By contrast, 13N-ammonia PET-MPI depicted scarred myocardium at follow-up in 38 (76%) patients. Of note, in one out of the five patients who did not present with any LGE depictable by CMR at follow-up, an area of reduced 13N-ammonia retention apparently corresponding to a myocardial scar was found in 13N-ammonia PET-MPI (Fig. 2).

**Fig. 3:**
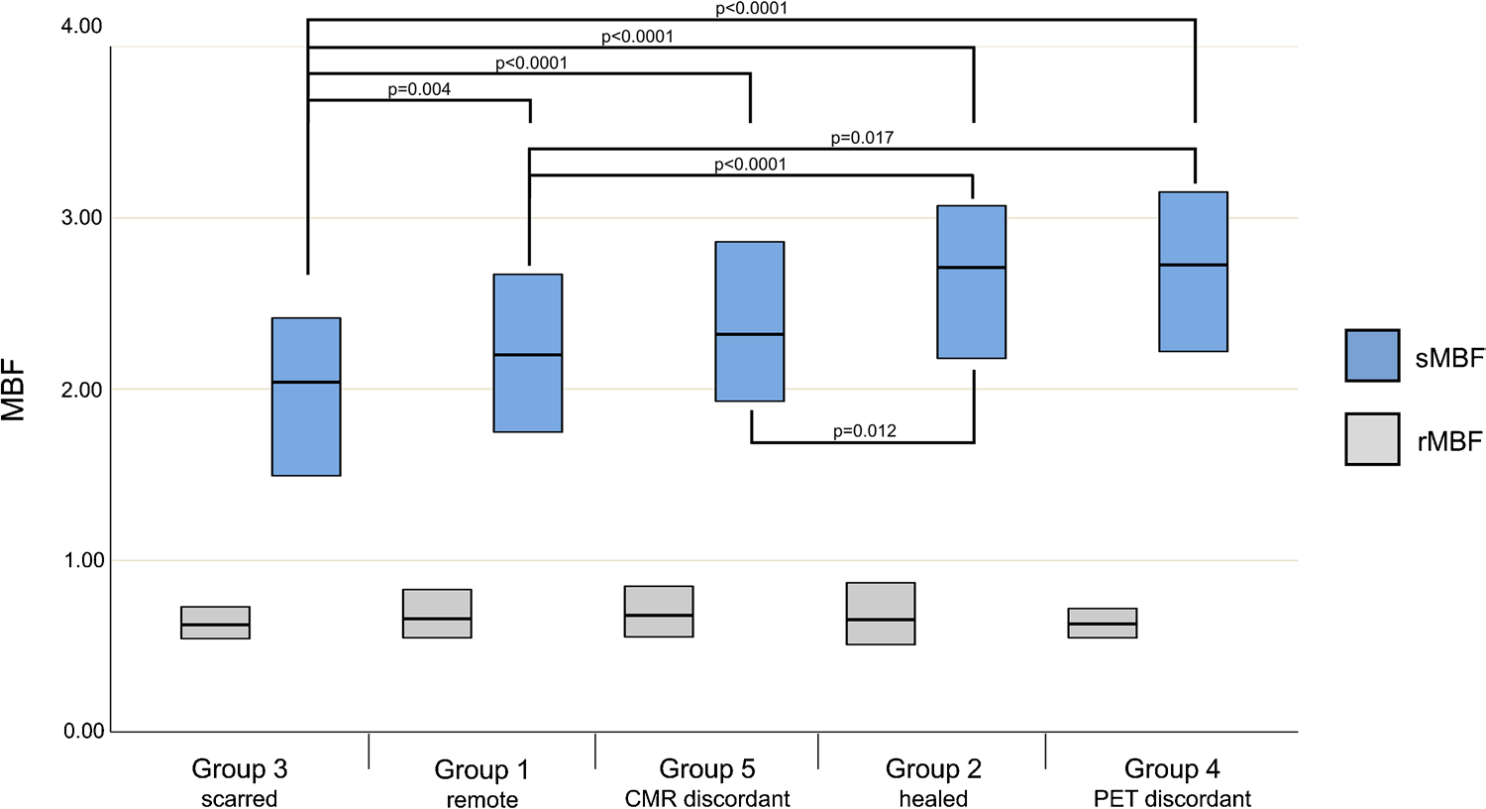
Boxplots of MBF values from rest (gray boxes) and stress (blue boxes) 13N-ammonia PET imaging stratified according to the different groups of segments. Boxes depict the interquartile range (25th to 75th quartile) and contain a horizontal line depicting median values. Statistically significant differences between values from different groups are indicated by horizontal lines with whiskers and the corresponding *p*-values

**Fig. 4:**
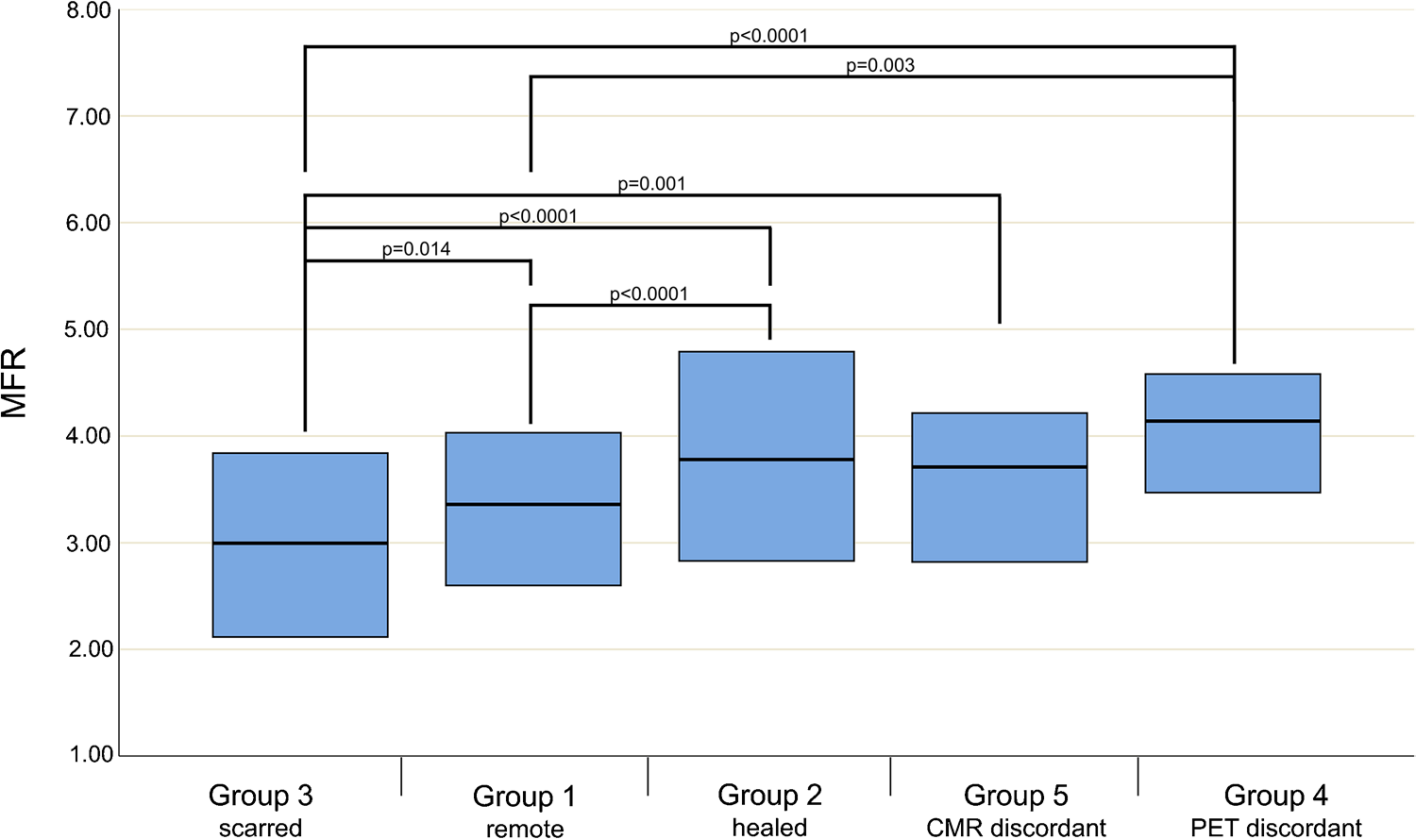
Boxplots of MFR values from 13N-ammonia PET imaging stratified according to the different groups of segments

**Fig. 5:**
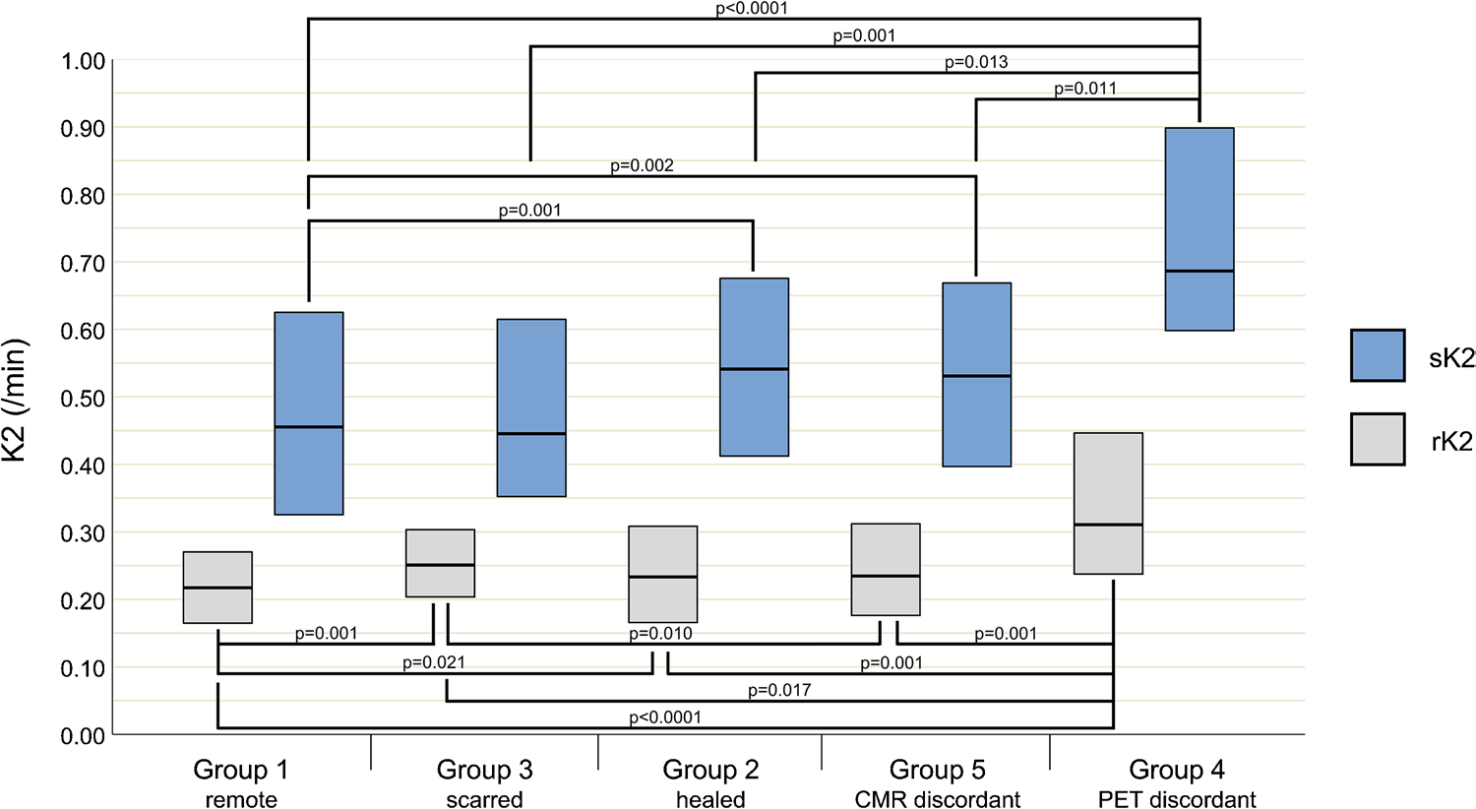
Boxplots of K2 values from rest (gray boxes) and stress (blue boxes) 13N-ammonia PET imaging stratified according to the different groups of segments

The distribution of segments among the pre-defined groups is displayed in Table 3. We found 19 (2.4%) segments among 9 patients, where 13N-ammonia PET-MPI revealed seemingly scarred myocardium, which could not be confirmed by simultaneously acquired CMR. Of these, 18 (94%) segments had initially presented with signs of inflammation at baseline and were therefore assigned to group 4, while in 1 (6%) remaining segment, no perceivable or measurable sign of inflammation could be found at baseline. Quantitative parameters as obtained from 13N-ammonia PET-MPI for all segments and stratified by groups, as well as the results from a comparison among groups, are given in Table 3 and visualized in Figs. 3, 4, and 5, while statistical details on the post hoc pairwise comparisons are provided in Table S1. While MBF at rest did not differ among the various groups, sMBF in segments from group 3 was significantly lower than in segments from all other groups. By contrast, sMBF in segments from groups 2 and 4 were significantly higher than in those in group 1, and, as a result, MFR in segments from groups 2 and 4 were also higher than in those in group 1. Finally, quantitative PET-MPI analysis revealed that the segments of group 4 showed a significantly higher 13N-ammonia washout rate K2 than the segments from all other groups, with an increase between + 24% and + 53%.

**Table 3:** Imaging findings per segment at baseline and at follow-up (*n* = 800)

	All segments (*n* = 800)	Group 1 Remote (*n* = 469 among 50 patients)	Group 2 Healed (*n* = 118 among 35 patients)	Group 3 Scarred (*n* = 72 among 33 patients)	Group 4 PET discordant (*n* = 18 among 9 patients)	Group 5 CMR discordant (*n* = 111 among 34 patients)	*p*-value
rMBF, mL_*_min^−1^_*_g^−1^	0.66 [0.55–0.83]	0.66 [0.55–0.83]	0.66 [0.51–0.88]	0.63 [0.54–0.73]	0.63 [0.54–0.74]	0.68 [0.55–0.85]	0.588
sMBF, mL_*_min^−1^_*_g^−1^	2.27 [1.84–2.79]	2.20 [1.75–2.68]	2.71 [2.18–3.08]†°	2.04 [1.48–2.42]†‡^+^°	2.73 [2.21–3.26]†	2.32 [1.93–2.88]	** 0.001**
MFR	3.43 [2.64–4.19]	3.36 [2.60–4.03]	3.78 [2.83–4.79]†*	3.00 [2.11–3.86]†	4.14 [3.45–4.61]†*	3.71 [2.74–4.22]*	** 0.001**
rK2, /min	0.23 [0.17–0.28]	0.22 [0.16–0.27]	0.24 [0.18–0.31]†	0.25 [0.20–0.30]†°	0.31 [0.24–0.46]†‡*°	0.21 [0.16–0.29]	** 0.001**
sK2, /min	0.49 [0.35–0.64]	0.46 [0.32–0.63]	0.53 [0.40–0.67]†	0.45 [0.35–0.62]	0.69 [0.59–0.92]†‡*°	0.52 [0.40–0.64]†	** 0.001**

Values given are median and IQR in square brackets. *p*-values indicate the significance level of the Kruskal-Wallis tests. Post hoc pairwise comparison with Benjamini-Hochberg adjustment for multiple comparisons indicates the following

^†^differs significantly from group 1

^‡^differs significantly from group 2

^*^differs significantly from group 3

^+^differs significantly from group 4

°differs significantly from group

**Fig. 6:**
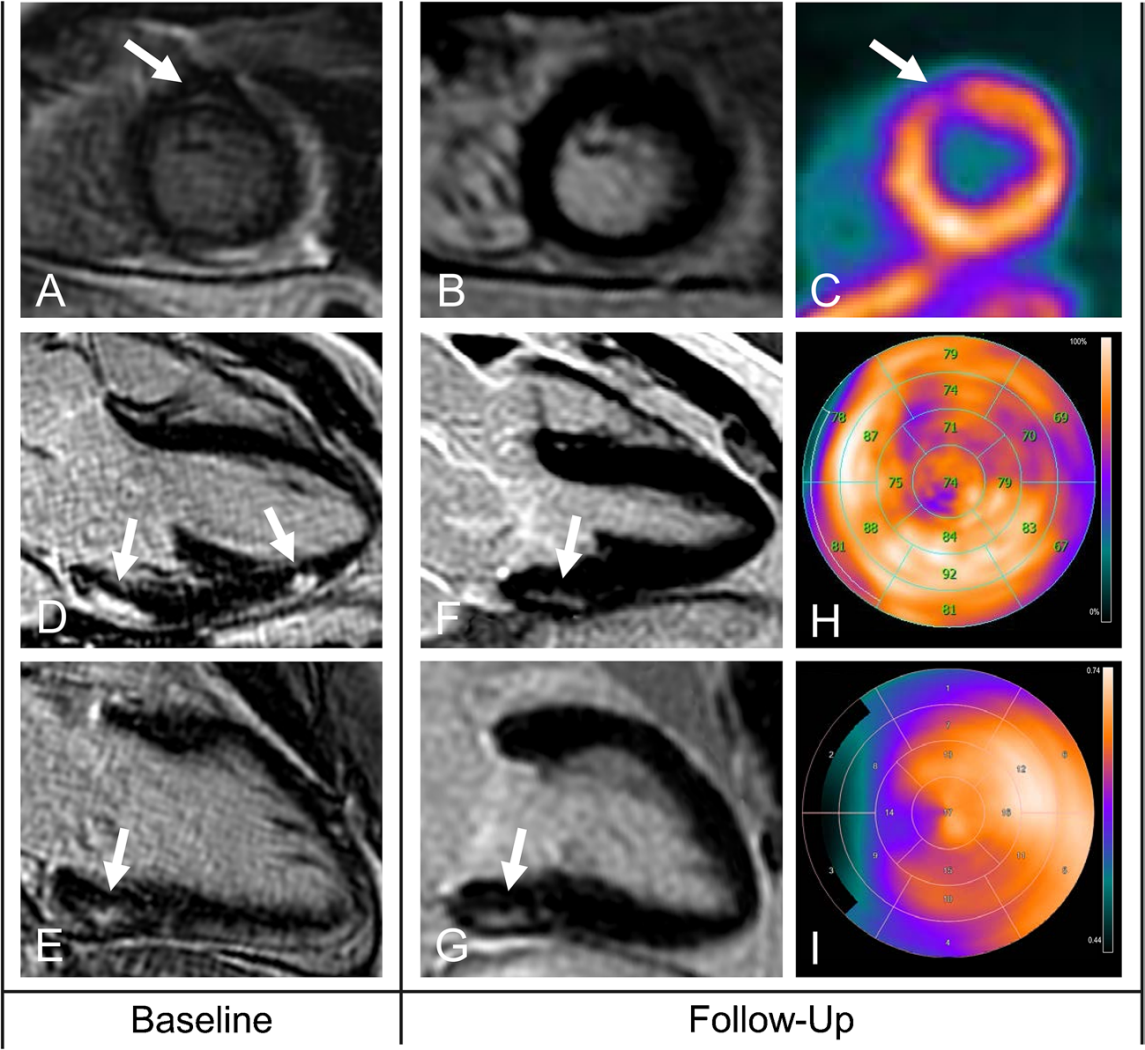
An imaging study of a 48-year-old male patient with a history of myocarditis diagnosed 7 years prior. CMR at baseline depicted a subtle area with mid-wall LGE in the anteroapical LV myocardium, as shown in the short-axis view (**A**). At follow-up, no LGE was depictable in this region (**B**), indicating the initial LGE to be due to inflammation. However, PET retention images showed reduced radionuclide retention in the same anteroapical region (**C**). Additional areas with subepicardial LGE at baseline, as shown in the three-chamber (**D**) and two-chamber (**E**) views were persistent at follow-up in the inferolaterobasal wall but not in the lateral wall (**F**, **G**). As depicted by the polar plots of PET retention data at follow-up (**H**), both areas showed reduced radionuclide retention. Finally, the polar plot depicting the 13N-ammonia washout rate (**I**) reveals increased values in the entire lateral and inferolateral wall extending to the anteroapical region

Interestingly, the PET discordant segments from group 4 clinically led to 10 (20%) patients in whom one or more areas of scar were diagnosed by 13N-ammonia PET-MPI but whereby no LGE was depictable by CMR. An example is provided in Fig. 6.

## Discussion

The main results of the present study may be summarized as follows:

1. Quantitative measurements of myocardial perfusion as obtained from 13N-ammonia PET-MPI remain altered in myocardial segments initially affected by inflammation in that these segments show higher hyperemic MBF and, consequently, MFR but also higher 13N-ammonia washout rates than remote segments.
2. At a median of 25 months after diagnosis of acute myocarditis, edema resolved in all patients, and apparent complete healing (i.e., absence of edema and LGE) was observed in 10% of patients.
3. In a small proportion of segments (2.4%) with signs of inflammation at baseline but apparent complete healing at follow-up, 13N-ammonia retention remained substantially reduced. On a patient level, this translated into 10 (20%) patients in whom one or more areas were diagnosed by 13N-ammonia PET-MPI as presenting with a myocardial scar.
4. The fact that the vast majority (94%) of these segments were found to be affected by inflammation at baseline and with 13N-ammonia washout rates increased by up to 53% in these segments as compared to remote segments suggests an association between inflammation at baseline and washout rates during follow-up.

CMR is recommended as an initial imaging modality in suspected myocarditis and has been shown to confer diagnostic and prognostic value in this disease entity [14, 16]. Hence, the current criteria for the diagnosis of myocarditis rely heavily on CMR findings, namely, the identification of myocardial edema and LGE [12]. As demonstrated by the present study, edema completely resolved in all patients 25 months after diagnosis. Moreover, LGE also resolved in 10% of patients, indicating complete healing. This finding and its proportion are in line with previous studies by Mahrholdt et al. [13] and Aquaro et al. [14], which documented the resolution of LGE in 26.7% (19/71) and 10.7% (20/187) of patients 6 months after the onset of symptoms. Hence, the present study corroborates these previous findings and lends further support to the evidence that initial LGE is not necessarily synonymous with irreversible myocardial damage. LGE may be caused by any process that leads to an enlargement of the interstitial space, thereby increasing the volume of distribution within the myocardium. During inflammation, this may be caused by inflammatory cell invasion, edema, and hyperemia [17].

The present work extends our knowledge beyond previous studies by addressing the long-term impact of inflammation on MBF. Through the combination of hybrid PET/MR technology and the implementation of a methodology that allows for the comparison of CMR and PET at a segmental level, our study provides unprecedented insights into the interplay between biomorphological findings from CMR and perfusion properties assessed at the molecular level by PET late after myocarditis. The importance of assessing the final status of myocardium on a regional (segmental) level rather than on a per-patient level is, in hindsight, corroborated by the fact that all patients in the present study exhibited at least one segment from each group.

Our first main finding reveals an association between the dynamics during and after myocarditis, resulting in myocardium arriving in various states. Segmental MBF at rest did not differ among the groups, likely due to the size of residual fibrosis in most patients being too small to exert any substantial effect in terms of MBF assessment. On the other hand, surprisingly, we found that, on a segmental level, hyperemic MBF and MFR were higher in segments initially affected by inflammation but with (apparent) complete healing at follow-up and in those with discordant findings between 13N-ammonia and CMR at follow-up as compared to remote segments not initially affected by inflammation. This finding contradicts the scarce literature suggesting reduced coronary flow reserve and possibly even ischemia in patients with a history of inflammation. Klein et al. found that in 29 patients with acute biopsy-proven inflammatory myocardial infiltrates, the coronary reserve, as assessed by an argon gas chromatographic method, was reduced as compared to a control group [11]. The explanation for the discrepancies between our findings and the study by Klein et al. is likely multi-layered. The main cause may be due to the fact that Klein et al. assessed patients at a very different state of the disease. It may be hypothesized that in the acute phase of myocarditis, the coronary microcirculation is involved in the inflammation process either via direct infection of the endothelium or via a cellular- or humoral-mediated immunopathologic process leading to altered vascular function and impaired vasodilator response and, ultimately, to myocyte damage. Our results indicate that the vasodilator response late after myocarditis is not only restored in healed myocardium but may even exceed that of remote myocardium. One potential explanation may be that myocardial inflammation mediates a local increase in the expression of A2A adenosine receptors. The latter have been suggested to mediate the anti-inflammatory actions of adenosine in a variety of cell types, including human monocytes [18], but whether such an upregulation also takes place in the myocardial microcirculation during inflammation and persists remains currently unknown. Hence, whether our finding is due to persistent structural alterations in the endothelium or adjacent myocytes, because of increased responsiveness to adenosine, or mediated through a yet unknown mechanism must be elucidated by future studies.

The second main finding of our study relies on the ability of the kinetic models used for the calculation of MBF to also provide additional information beyond MBF, for example, in the form of the radionuclide washout rate. The washout rate in PET has been identified as a surrogate marker for myocyte metabolic and membrane integrity in the setting of ischemic cardiomyopathy for different radiotracers [19, 20]. Our study suggests an association between myocardial inflammation and increased 13N-ammonia leakage, potentially through impairment of myocyte membrane integrity or increased transendothelial permeability. This could explain the findings from previous studies reporting perfusion abnormalities on SPECT MPI in the setting of acute myocarditis [6, 7] and is supported by a recent study documenting increased regional SPECT tracer washout in patients with cardiac sarcoidosis and inflammation as evidenced by 18F-fluorodeoxyglucose PET imaging [21]. The fact that in the present study, 94% of the PET discordant segments initially showed inflammation makes it reasonable to assume that this cannot be explained by a simple misclassification by PET. However, the exact biomorphological correlate underlying the increased washout cannot be comprehensively clarified from our observational study and remains to be elucidated by future investigation.

Our findings also suggest that the common definition of complete healing after myocarditis, currently based on the complete resolution of myocardial edema and LGE, along with normalized biomarkers, may not be completely comprehensive and does not necessarily reflect true restitutio ad integrum. However, it remains speculative as to why myocardium presents with long-term alterations in some patients but not in others and whether our findings have any prognostic implications. While the results of the present study ultimately remain hypothesis-generating in that regard, the fact that a substantial number of patients presented with PET findings corresponding to myocardial scar tissue in the absence of any substrate on concomitant CMR nevertheless has immediate implications on clinical imaging routine because our results indicate that discordant findings between CMR and PET-MPI (and potentially also in SPECT-MPI) in patients with a history of myocarditis and/or inflammation should not readily be interpreted as false-positive (or false-negative) but may indeed be the result of subtle post-inflammatory sequelae on a cellular level not depictable with current CMR techniques. Our findings suggest that future studies, ideally combining molecular and morphological imaging as well as humoral markers, are needed to improve our understanding of the long-term effects of inflammation on the myocardium and the potential implications for our patients.

## Conclusions

In patients with a history of myocarditis, quantitative measurements of myocardial perfusion as obtained from PET-MPI remain altered in areas initially affected by inflammation. In a minority of patients, increased washout, arguably induced by inflammation, translates into PET findings, namely, areas with apparent scar but without a corresponding substrate on CMR.

## Study limitations

The main limitation of this study is the absence of PET/MR data at baseline, precluding us from a statement on changes in quantitative myocardial blood flow parameters between baseline and parameters. Unfortunately, reliable T1- and T2-mapping techniques for the PET/MR device were not available at the time of enrollment. Furthermore, it may be seen as a limitation of the study design that several different MR devices, including both 1.5 and 3.0 Tesla devices, were used for baseline imaging, potentially introducing differences in diagnostic accuracy, particularly for LGE detection. However, we aimed to mitigate this issue by performing a thorough and careful re-analysis of all baseline CMR scans, performing side-by-side visual comparisons, and applying a standardized approach by using only one software tool for analysis and by only including segments with LGE at least 5 standard deviations over remote myocardium for both the baseline and follow-up CMR scans. While this semi-automated thresholding should have improved specificity, potentially at the cost of slightly lower sensitivity, this methodology should have equaled out potential sensitivity issues among different scanners. Of note, all patients in the present study exhibited at least one segment from each group, and the contribution of segments to a group varied strongly per patient (Table 3). Importantly, however, MBF and K2 values did not differ significantly among patients contributing only one segment versus those contributing more segments, rendering it unlikely that those patients with exceptionally high or low values for any given parameter were, by chance, those who contributed the most segments to a given group. Finally, we acknowledge that the segmental comparison between PET and CMR may not have been perfect due to the inherent technical differences in data acquisition and reader-related variabilities in image alignment. This may potentially have led to undetected correlations and differences in the statistical analysis process.

### Supplementary Information

Below is the link to the electronic supplementary material.

Supplementary file1 (DOCX 15 KB)

## Data Availability

The datasets generated during and/or analyzed during the current study are available from the corresponding author upon reasonable request.
