# Supplementary material for: Long-term impact of myocardial inflammation on quantitative myocardial perfusion—a descriptive PET/MR myocarditis study
Source: Eur J Nucl Med Mol Imaging. 2023 Jul 1;50(12):3609–18. doi: 10.1007/s00259-023-06314-0 (PMC10547808; doi:10.1007/s00259-023-06314-0)
Supplement: Supplementary file 1 — Supplementary file1 (DOCX 15 KB) [file 259_2023_6314_MOESM1_ESM.docx]

**Table S1.** Detailed results of pairwise comparison between groups

| **Variable** | Comparison | p-value | k-value |
| --- | --- | --- | --- |
| rMBF | n/a | n/a | n/a |
| sMBF | **3-2** | **<0.0001** | 0.0050 |
|  | **1-2** | **<0.0001** | 0.0050 |
|  | **3-5** | **<0.0001** | 0.0050 |
|  | **3-4** | **<0.0001** | 0.0050 |
|  | **3-1** | **0.004** | 0.0250 |
|  | **5-2** | **0.012** | 0.0300 |
|  | **1-4** | **0.017** | 0.0350 |
|  | 1-5 | 0.052 | 0.0400 |
|  | 5-4 | 0.146 | 0.0450 |
|  | 2-4 | 0.879 | 0.0500 |
| MFR | **3-2** | **<0.0001** | 0.0050 |
|  | **1-2** | **<0.0001** | 0.0050 |
|  | **3-4** | **<0.0001** | 0.0050 |
|  | **3-5** | **0.001** | 0.0200 |
|  | **1-4** | **0.003** | 0.0250 |
|  | **3-1** | **0.014** | 0.0300 |
|  | 5-4 | 0.040 | 0.0350 |
|  | 5-2 | 0.077 | 0.0400 |
|  | 1-5 | 0.083 | 0.0450 |
|  | 2-4 | 0.254 | 0.0500 |
| rK2 | **1-4** | **<0.0001** | 0.0050 |
|  | **5-4** | **<0.0001** | 0.0050 |
|  | **1-3** | **0.001** | 0.0150 |
|  | **2-4** | **0.001** | 0.0150 |
|  | **5-3** | **0.010** | 0.0250 |
|  | **3-4** | **0.017** | 0.0300 |
|  | **1-2** | **0.021** | 0.0350 |
|  | 5-2 | 0.118 | 0.0400 |
|  | 2-3 | 0.221 | 0.0450 |
|  | 1-5 | 0.764 | 0.0500 |
| sK2 | **1-4** | **<0.0001** | 0.0050 |
|  | **1-2** | **0.001** | 0.0100 |
|  | **3-4** | **0.001** | 0.0100 |
|  | **1-5** | **0.002** | 0.0200 |
|  | **5-4** | **0.011** | 0.0250 |
|  | **2-4** | **0.013** | 0.0300 |
|  | 3-2 | 0.113 | 0.0350 |
|  | 3-5 | 0.153 | 0.0400 |
|  | 1-3 | 0.403 | 0.0450 |
|  | 5-2 | 0.874 | 0.0500 |

k-values indicate the significance level adjusted by the Benjamini-Hochberg procedure. Bold indicates statistically significant pairwise comparisons with a p-value <0.05 and below the adjusted significance level.

*rMBF = rest myocardial blood flow, sMBF = stress myocardial blood flow, MFR = myocardial flow reserve, rK2 = rest K2 (washout rate), sK2 = stress K2 (washout rate)*
